# Sweat rate of highly trained junior rowers during a session of low intensity endurance training

**DOI:** 10.1186/2052-1847-7-S1-O11

**Published:** 2015-08-11

**Authors:** Ilka Popp, Gunnar Treff, Jürgen M Steinacker

**Affiliations:** 1 Division of Sports and Rehabilitation Medicine, University of Ulm, Ulm, Germany

## Background

Hypohydration is associated with impaired endurance performance and increased heat stress. Therefore, rehydration after exercise needs to be sufficient. However, several athletes tend to hypohydrate especially during training camps. To allow exact quantification of necessary rehydration volume in order to prevent hypohydration, this study aimed to determine the sweat rate of highly trained junior rowers during a session of low intensity rowing training.

## Methods

Eight highly trained junior rowers (M8+) participated in the study (age: 17.5 ± 0.5 yrs; body mass (BM): 89.5 ± 4.5 kg; height: 193.1 ± 2.9 cm; body surface area (BSA): 2.2 ± 0.1 m^2^; VO_2max_: 64.2 ± 5.5 ml/kg/min).

Haematocrit in capillary blood as well as the urine specific gravity (USG) were measured in the morning at fasting state as a measure of hydration status.

BM was measured before (PRE) and directly after (POST) a session of low intensity rowing (20 km; 1.5 hours). Athletes dried themselves with a towel before weighing and wore dry underpants only. Water consumption during training was recorded. Sweat rate was calculated as follows:

$$\text{Sweat}\;\text{rate}(\text{l}/\text{h})=\frac{BM_{PRE}-BM_{POST}-consumed\;water}{90\;min}\times 60\;min$$

The environmental conditions were 16.5 °C and cloudy.

## Result

Haematocrit values before training were 50 ± 2.6 % and USG was 1.022 ± 0.005. BM loss during the complete training session was 0.7 ± 0.5 kg. Athletes consumed 0.8 ± 0.3 l of water. The sweat rate amounted to 0.96 ± 0.26 l/h equal to 0.44 ± 0.12 l/h/m^2^. Highest sweat rate amounted to 1.30 l/h.

## Discussion

Pretest data indicated euhydration in 6 athletes, however in 2 athletes signs of mild hypohydration were detected, what possibly influenced the results.

The results demonstrate a relevant loss of body water through sweat and respiration during a single session of low intensity exercise even at moderate temperatures. In hot environment, higher sweat loss has to be expected. As data do not reflect urine and fecal body water loss, total body water loss exceeds sweat rate. This has to be taken into account when calculating sufficient rehydration volume.

## Conclusion

Sweat rate of highly trained junior rowers ranges from 0.55 to 1.30 l/h in a moderate environment. Athletes and coaches should be aware of water loss to ensure sufficient rehydration during and after exercise. Furthermore, monitoring of hydration status is recommended in high performance sports.

